# Case report: Concurrent intravestibular schwannoma mimicking Ménière's disease and cochlear hydrops detected *via* delayed three-dimensional fluid-attenuated inversion recovery magnetic resonance imaging

**DOI:** 10.3389/fneur.2022.1043452

**Published:** 2022-11-09

**Authors:** Koji Nishimura, Toshihisa Murofushi, Nobuhiro Hakuba

**Affiliations:** Department of Otolaryngology, Teikyo University School of Medicine, Mizonokuchi Hospital, Kawasaki, Japan

**Keywords:** delayed 3D-FLAIR MR imaging, endolymphatic hydrops (EH), intralabyrinthine schwannomas (ILSs), intravestibular schwannoma, vestibular evoked myogenic potential (VEMP), video head impulse test (vHIT), Ménière's disease

## Abstract

**Objective:**

To present a case of intralabyrinthine schwannoma (ILS) presenting as Ménière's disease diagnosed *via* 4-h delayed gadolinium-enhanced three-dimensional fluid-attenuated inversion recovery magnetic resonance imaging (3D-FLAIR MRI) and treated successfully using the translabyrinthine approach.

**Patient:**

A patient who was diagnosed with intravestibular ILS.

**Interventions:**

The patient underwent comprehensive preoperative neurological examinations and MRI. The tumor was resected using the translabyrinthine approach and was pathologically confirmed as schwannoma based on the surgical specimen.

**Main outcome measures:**

Preoperative audiogram and vestibular test findings and MRI images.

**Results:**

Preoperatively, pure-tone audiogram showed progressive sensorineural hearing loss only on the affected side. The video head impulse test and vestibular evoked myogenic potential test showed vestibular dysfunction on the affected ear. Immediate gadolinium-enhanced T1-weighted MRI revealed an enhanced region in the vestibule. Meanwhile, magnetic resonance cisternography showed a filling defect. Delayed 3D-FLAIR MRI revealed a signal void in the scala media of the cochlea indicative of cochlear hydrops, and a strong signal in the perilymph at the basal cochlea suggestive of impaired blood–labyrinthine barrier.

**Conclusion:**

Delayed 3D-FLAIR MRI is useful in diagnosing concurrent ILSs and endolymphatic hydrops.

## Introduction

Vestibular schwannomas (VSs) are benign neoplasms commonly arising from the Schwann cells of the vestibular division of the vestibulocochlear nerve. Intralabyrinthine schwannomas (ILSs) are a rare clinical entity, and they arise from the distal branches of the vestibular and cochlear nerves within the membranous labyrinth (1, 2). The common symptoms of ILSs are unilateral hearing loss, tinnitus, imbalance/dizziness and occasionally episodes of vertigo, which are similar to those of Ménière's disease (MD) (3, 4). Endolymphatic hydrops is a characteristic of MD and other inner ear diseases, such as endolymphatic tumors. Moreover, VSs can cause endolymphatic hydrops (5–9). Naganawa et al. developed magnetic resonance imaging (MRI) methods to facilitate the visualization of endolymphatic hydrops in living humans using intravenous gadolinium-based contrast media (10, 11). Herein, we report a case of concurrent ILS mimicking MD and cochlear hydrops diagnosed *via* gadolinium-enhanced MRI.

## Case report

**Figure 1:**
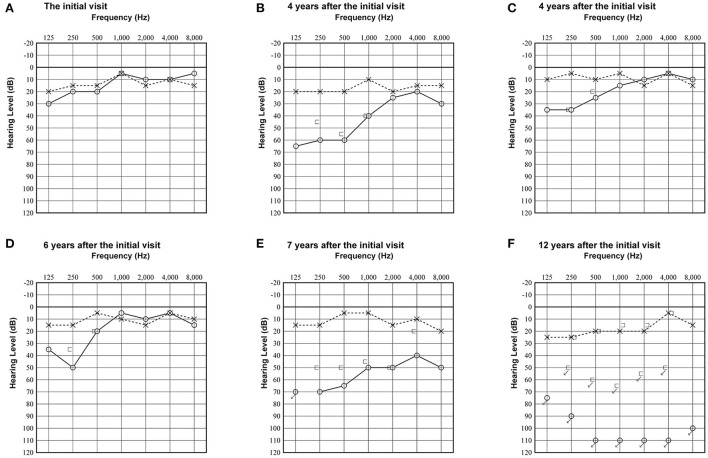
Pure-tone audiogram at the initial visit showed almost normal hearing threshold on both sides **(A)**. Audiogram 4 years after the initial visit revealed moderate sensorineural hearing loss in the right ear at low and middle frequencies **(B)**. Audiogram after treatment with corticosteroid with partial hearing improvement **(C)**. Audiogram 6 years after the initial visit showed mild hearing loss in the right ear only at low frequencies **(D)**. Audiogram 7 years after the initial visit revealed severe hearing loss in the right ear at all frequencies **(E)**. Audiogram 12 years after the initial visit showed profound sensorineural hearing loss in the right ear **(F)**.

**Figure 2:**
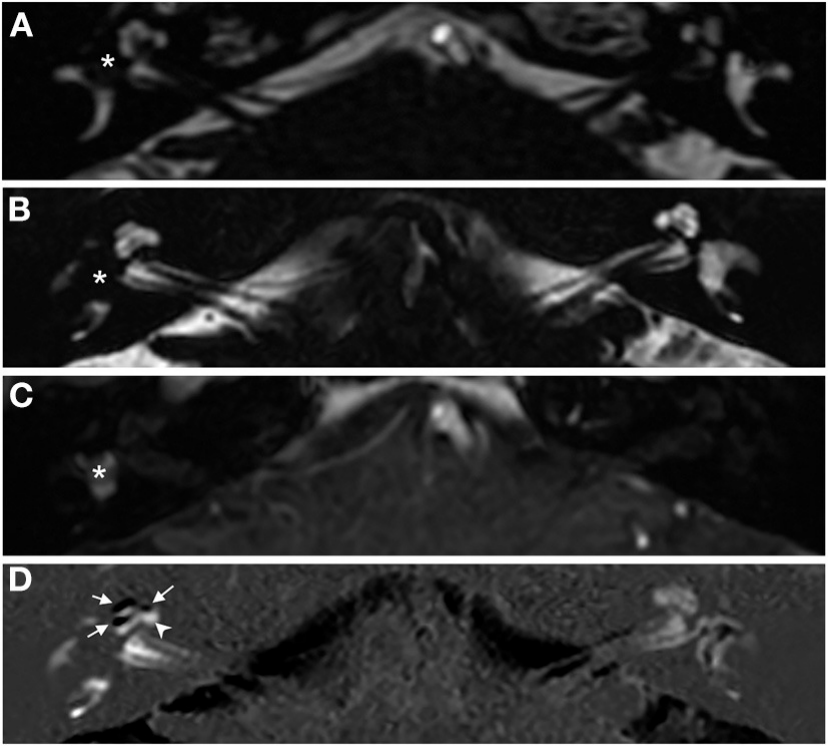
Axial magnetic resonance (MR) images obtained from a patient with concurrent right intravestibular schwannoma and endolymphatic hydrops in the right cochlea. **(A)** MR cisternography images obtained 4 years after the patient's initial visit to our department. **(B–D)** MR images obtained 12 years after the patient's initial visit to our department. **(A,B)** MR cisternography revealed a filling defect in the right vestibule (asterisks). **(C)** Gadolinium-enhanced T1-weighted imaging showed an enhanced region in the right vestibule indicating the presence of a hypervascular mass (asterisk). **(D)** Delayed 3D-FLAIR image, subtraction of the positive endolymphatic image from the positive perilymphatic image revealed black areas (arrowheads) representing the right cochlear hydrops and diffuse perilymphatic hyperintense area (arrowhead) indicative of blood–labyrinth barrier impairment.

**Figure 3:**
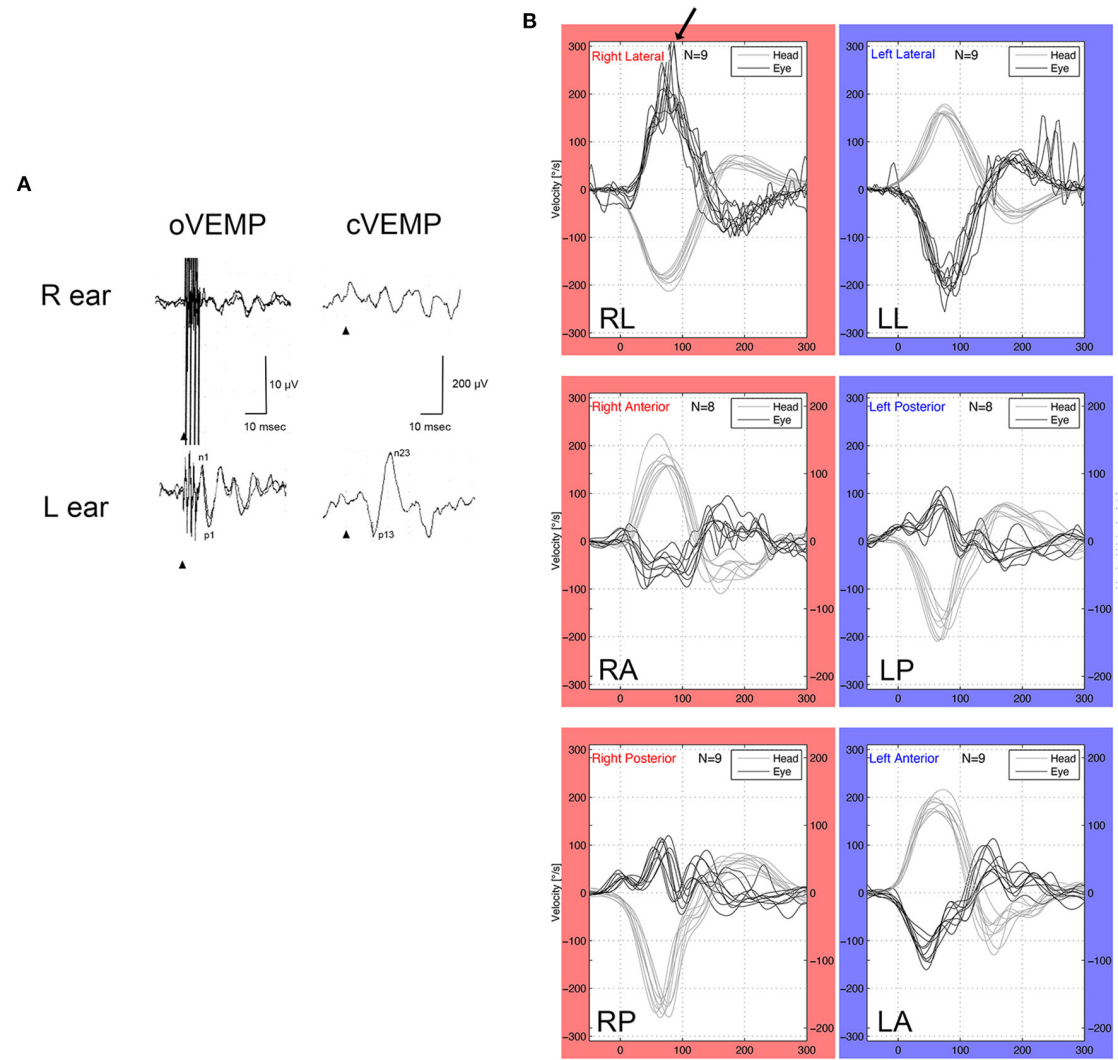
VEMP and vHIT. **(A)** oVEMP (500-Hz bone conduction) and cVEMP (500-Hz air condition). The patient had normal oVEMP and cVEMP on the unaffected side (left ear), and she presented with abnormal oVEMP and cVEMP in the affected side (right ear). **(B)** vHIT showed hyporeflexia in the anterior and posterior semicircular canals of both ears. Meanwhile, vHIT of the left lateral semicircular canal was normal, and vHIT of the right lateral semicircular canal had covert saccades (arrow), which indicated that the right lateral semicircular canal had decreased function.

**Figure 4:**
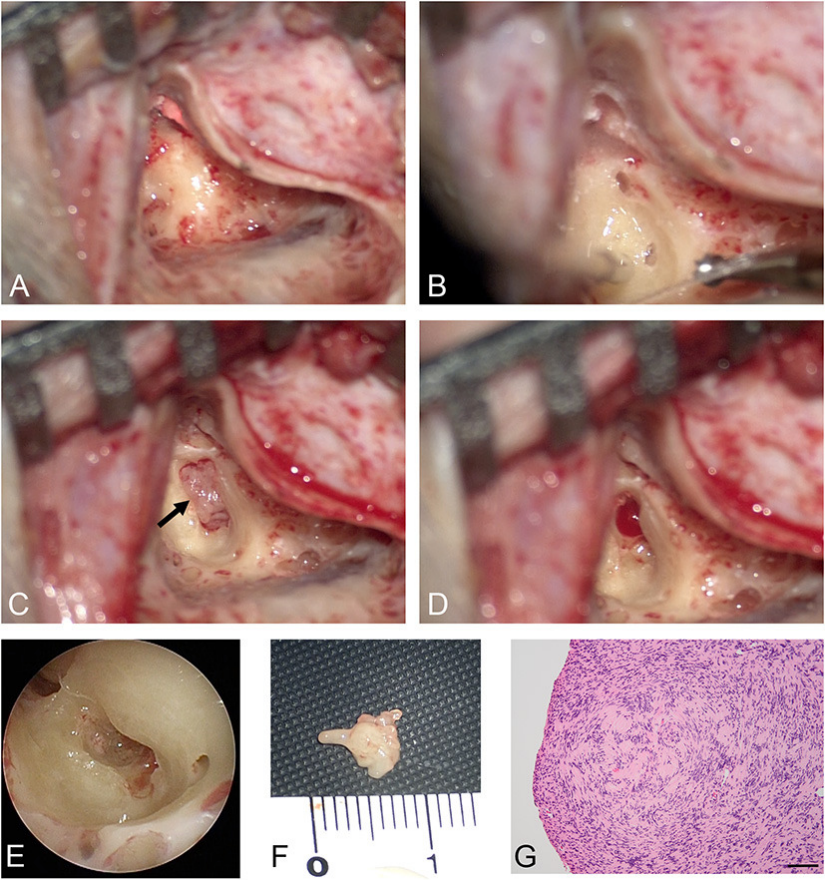
Intraoperative findings. After cortical mastoidectomy **(A)** and labyrinthectomy **(B)**, a pink mass was observed in the right vestibule (arrow) **(C)**. After tumor resection, the intravestibular region was evaluated both microscopically **(D)** and endoscopically **(E)**. An 8-mm mass was excised **(F)**. **(G)** High-power microphotograph showed a nuclear palisading pattern. Scale bar = 100 μm.

A woman in her 30's visited our department due to hyperacusis in the right ear. Audiogram showed almost normal hearing level (Figure 1A). The patient did not present with spontaneous or positional nystagmus. However, she occasionally experienced vertigo in the morning 4 years prior to her initial visit to our department. Four years after the first visit, she presented with vertigo, tinnitus, and hearing loss in the right ear. Audiogram revealed moderate right sensorineural hearing loss particularly at low frequencies (Figure 1B). Magnetic resonance (MR) cisternography showed no VS in the cerebellopontine angle. However, a filling defect of fluid intensity was detected in the vestibule of the right ear, and this finding was overlooked by an otolaryngologist and a radiologist (Figure 2A asterisk). Corticosteroid was prescribed for 7 days for acute sensorineural hearing loss in the right ear. Thereafter, partial hearing improvement was observed (Figure 1C). Nevertheless, the patient continually presented with tinnitus in the right ear and dizziness. Thus, her hearing function was evaluated regularly. Results showed moderate hearing loss in the right ear at low frequencies (Figure 1D). Seven years after the initial visit, she complained of worsening hearing loss in the right ear. Audiogram revealed moderate sensorineural hearing loss at all frequencies in the right ear (Figure 1E). She was again treated with corticosteroid. However, there was no hearing improvement. Twelve years after the initial visit, her hearing function deteriorated. Audiogram revealed profound sensorineural hearing loss in the right ear (Figure 1F). Both cervical and ocular vestibular evoked myogenic potential (cVEMP and oVEMP) were not detected upon right ear stimulation (Figure 3A). The video head impulse test (vHIT) showed decreased vestibulo-ocular reflex (VOR) gains in both the anterior and posterior canals on the affected and unaffected sides. Covert catch-up saccades were observed during eye movement elicited by right lateral semicircular canal stimulation. This result indicated decreased right lateral semicircular canal function (Figure 3B). Due to episodes of recurring vertigo and fluctuating hearing loss, endolymphatic hydrops, possibly correlated with MD, was considered as the preliminary diagnosis (12). Thus, delayed 3D-FLAIR MRI was performed to confirm endolymphatic hydrops (10, 11) or identify other conditions. MR cisternography showed a filling defect of fluid intensity in the vestibule of the right ear (Figure 2B asterisk). Gadolinium-enhanced T1-weighted imaging revealed a moderately enhanced region in the right vestibule, which indicated a hypervascular mass in the right vestibule (Figure 2C asterisk). Delayed 3D-FLAIR MRI showed endolymphatic hydrops in the right cochlea (Figure 2D arrows) with a stronger signal intensity in the scala tympani in the basal portion of the cochlea (Figure 2D arrowhead) than in the contralateral ear. The patient wanted to undergo intralabyrinthine tumor resection. Thus, surgery using the translabyrinthine approach was performed (Figure 4). Wide mastoidectomy was performed, followed by transmastoid labyrinthectomy. Then, a pink irregular shaped mass was found to fill the vestibule. After tumor resection, residual tumor was not observed microscopically and endoscopically. An 8-mm mass was completely resected. The whorls of spindle-shaped cells and the palisading structure of cells were consistent with schwannoma. The patient did not present with complications nor vertigo after the surgery. Further, she was followed-up every month, and she underwent clinical vestibular evaluations.

## Discussion

Herein, we present a case of concurrent endolymphatic hydrops diagnosed *via* 3D-FLAIR MRI and ILS diagnosed *via* pathological examination of surgical specimen. To the best of our knowledge, there are only two studies describing endolymphatic hydrops diagnosed *via* delayed 3D-FLAIR MRI in patients with ILS (7, 13). One possible mechanism underlying endolymphatic hydrops is the presence of a tumor obstructing the ductus reuniens, which connects the cochlear duct and the saccule. Endolymphatic hydrops is also detected *via* delayed 3D-FLAIR MRI (14) and non-contrast-enhanced 3D-FLAIR MRI (6) in some VS cases. Hence, other than mechanical stenosis of the ductus reuniens, a common mechanism associated with this condition may exist between VSs and ILSs.

ILSs commonly cause unilateral hearing loss, tinnitus, vertigo, and aural fullness, which fairly overlap with MD symptoms. In accordance with this finding, 39% of patients with ILS were previously diagnosed with MD (15). If a patient is diagnosed with MD, the diagnosis is more challenging to reconsider. Our patient was also misdiagnosed with MD. However, she underwent gadolinium-enhanced T1-weighted MRI, which revealed a vestibular tumor. Prior to the initial series of ILSs diagnosed *via* MRI (16), ILS was diagnosed incidentally during surgery (17, 18), or autopsy (19, 20). Currently, MRI is the gold standard for diagnosing ILSs (1, 2). ILSs are characterized a low signal intensity within the membranous labyrinth, which appears as filling defects, on thin-section T2-weighted images. On post-gadolinium T1-weighted images, ILSs appear as enhanced masses. The imaging characteristics of our patient were in accordance with those of ILSs. Delayed 3D-FLAIR MRI was initially performed to visualize endolymphatic hydrops in patients with MD (10, 11, 21). A few studies have reported the characteristics of ILSs on delayed 3D-FLAIR MRI (7, 13, 22). The strong enhancement in the ipsilateral cochlear basal on delayed 3D-FLAIR MRI in the current case was in accordance with that of two previous reports on perilymphatic hyperintense areas surrounding the tumor (13, 22). Perilymphatic enhancement on delayed 3D-FLAIR MRI was also detected in patients with intracanalicular or cerebellopontine angle VSs, which might be caused by leakage of gadolinium into the cochlear perilymphatic space *via* the impaired blood–labyrinth barrier (14). Thus, sensorineural hearing loss in a subset of patients with VSs and ILSs is caused by alterations in blood–labyrinth barrier permeability. In addition, hearing loss was the most common presenting symptom in patients with ILSs regardless of tumor location (2). Homann et al. first detected endolymphatic hydrops *via* delayed 3D-FLAIR MRI in a patient with ILS (7). In similar studies investigating ILSs *via* delayed 3D-FLAIR MRI, endolymphatic hydrops was detected in 11.1% (3/27) (13), and 0% (0/3) of patients (22), respectively. However, in studies about ILSs detected *via* non-contrast-enhanced 3D-FLAIR MRI, endolymphatic hydrops was observed in 46.7% (14/30) of patients (23). Delayed 3D-FLAIR MRI is more effective in diagnosing endolymphatic hydrops than non-contrast-enhanced 3D-T2-weighted gradient-echo steady-state sequences (FIESTA-c) particularly in cases without ILSs (24). Of note, delayed 3D-FLAIR MRI in cases of ILSs might underestimate endolymphatic hydrops since gadolinium might leak in the endolymph due to impairment of the inner ear microenvironment caused by the tumor. Thus, different modalities can affect the assessment of endolymphatic hydrops in ILSs. In our case, concurrent endolymphatic hydrops and intravestibular ILS were detected *via* delayed 3D-FLAIR MRI. Endolymphatic hydrops may exist with ILSs. However, it cannot be clearly identified *via* delayed 3D-FLAIR MRI.

In the current case, the asymmetric ratios of cVEMP and oVEMP were 100%, which indicated severe otolith dysfunction on the affected side. Approximately 75% of patients with intravestibular ILS and 55.6% of patients with intracochlear ILS present with an abnormal cVEMP (25). Hence, unlike auditory functional differences, intralabyrinthine localizations could affect vestibular functional differences. Our patient presented with decreased VOR gains in both sides of the anterior and posterior semicircular canals, and covert catch-up saccades in the lateral semicircular canal on the affected side. The long duration of unilateral vestibular dysfunction (16 years in the current case) might help achieve good dynamic visual performance. Lee et al. presented a case of ILS mimicking MD based on a positive vHIT result in all semicircular canals on the affected side (26). The causes of decreased VOR gains on the left anterior and posterior semicircular canals remain unknown. However, the risk of ILS should be considered.

Hearing function cannot be preserved with surgical resection of ILSs. Thus, in patients with serviceable hearing or tolerable symptoms, the wait-and-see strategy is recommended (1, 8). Surgery is indicated if ILS extends toward the cerebellopontine angle or into the middle ear, or if a patient presents with intractable vestibular symptoms (1). In cases in which surgery can be performed, tumor localization is important in surgical planning. In the current case, the tumor was located in the vestibule and did not extend to other regions. Hence, according to the classification of Kennedy (1) or Salzman (2), it was considered an intravestibular type.

## Conclusion

Combined fluctuating hearing loss and episodic vertigo attacks can be observed in ILS and MD. Therefore, delayed 3D-FLAIR MRI is useful in the diagnosis of concurrent ILSs and endolymphatic hydrops.

## Data availability statement

The raw data supporting the conclusions of this article will be made available by the authors, without undue reservation.

## Ethics statement

Ethical review and approval was not required for the study on human participants in accordance with the local legislation and institutional requirements. The patients/participants provided their written informed consent to participate in this study. Written informed consent was obtained from the individual(s) for the publication of any potentially identifiable images or data included in this article.

## Author contributions

KN conceived the idea of the study and drafted the original manuscript. All authors reviewed the manuscript draft, revised its intellectual content critically and approved the final version of the manuscript for publication.

## Funding

The study was supported by JSPS KAKENHI Grant Number 22H03239.

## Conflict of interest

The authors declare that the research was conducted in the absence of any commercial or financial relationships that could be construed as a potential conflict of interest.

## Publisher's note

All claims expressed in this article are solely those of the authors and do not necessarily represent those of their affiliated organizations, or those of the publisher, the editors and the reviewers. Any product that may be evaluated in this article, or claim that may be made by its manufacturer, is not guaranteed or endorsed by the publisher.
